# The *AMT1* Arginine Methyltransferase Gene Is Important for Plant Infection and Normal Hyphal Growth in *Fusarium graminearum*

**DOI:** 10.1371/journal.pone.0038324

**Published:** 2012-05-31

**Authors:** Guanghui Wang, Chenfang Wang, Rui Hou, Xiaoying Zhou, Guotian Li, Shijie Zhang, Jin-Rong Xu

**Affiliations:** 1 College of Plant Protection, Northwest A&F University, Yangling, Shaanxi, China; 2 Department of Botany and Plant Pathology, Purdue University, West Lafayette, Indiana, United States of America; Seoul National University, Republic of Korea

## Abstract

Arginine methylation of non-histone proteins by protein arginine methyltransferase (PRMT) has been shown to be important for various biological processes from yeast to human. Although PRMT genes are well conserved in fungi, none of them have been functionally characterized in plant pathogenic ascomycetes. In this study, we identified and characterized all of the four predicted PRMT genes in *Fusarium graminearum*, the causal agent of Fusarium head blight of wheat and barley. Whereas deletion of the other three PRMT genes had no obvious phenotypes, the Δ*amt1* mutant had pleiotropic defects. *AMT1* is a predicted type I PRMT gene that is orthologous to *HMT1* in *Saccharomyces cerevisiae*. The Δ*amt1* mutant was slightly reduced in vegetative growth but normal in asexual and sexual reproduction. It had increased sensitivities to oxidative and membrane stresses. DON mycotoxin production and virulence on flowering wheat heads also were reduced in the Δ*amt1* mutant. The introduction of the wild-type *AMT1* allele fully complemented the defects of the Δ*amt1* mutant and Amt1-GFP fusion proteins mainly localized to the nucleus. Hrp1 and Nab2 are two hnRNPs in yeast that are methylated by Hmt1 for nuclear export. In *F. graminearum*, *AMT1* is required for the nuclear export of FgHrp1 but not FgNab2, indicating that yeast and *F. graminearum* differ in the methylation and nucleo-cytoplasmic transport of hnRNP components. Because *AMT2* also is a predicted type I PRMT with limited homology to yeast *HMT1*, we generated the Δ*amt1* Δ*amt2* double mutants. The Δ*amt1* single and Δ*amt1* Δ*amt2* double mutants had similar defects in all the phenotypes assayed, including reduced vegetative growth and virulence. Overall, data from this systematic analysis of PRMT genes suggest that *AMT1*, like its ortholog in yeast, is the predominant PRMT gene in *F. graminearum* and plays a role in hyphal growth, stress responses, and plant infection.

## Introduction

In eukaryotic organisms, reversible phosphorylation of proteins by protein kinase and phosphatase is well known to regulate various growth and development processes. Protein methylation is another form of post-translational modifications that also play regulatory roles in various processes, including nucleo-cytoplasmic transport of proteins, transcriptional activation and elongation, mRNA precursors splicing, and signal transduction [1], [2], [3], [4]. The majority of protein methylation occurred at the arginine residues are catalyzed by protein arginine methyltransferases (PRMTs), which are divided into four major classes. Type I and type II PRMTs catalyze asymmetric and symmetric ω N^G^, N^G^-dimethylation of arginine residues, respectively [2]. Whereas type III PRMTs catalyze ω N^G^ monomethylation of arginines, type IV PRMTs catalyze the formation of δ N^G^-monomethylarginine. In human, type I PRMTs include *PRMT1*, *PRMT3*, *PRMT4*, *PRMT6*, and *PRMT8*. *PRMT5*, *PRMT7*, and *PRMT9* are type II PRMTs [5]. Whereas *PRMT1*, *PRMT3*, and *PRMT5* are well conserved in eukaryotic organisms, *PRMT2*, *PRMT8*, and *PRMT9* lack distinct orthologs in unicellular eukaryotes and may be required for tissue-specific functions in multicellular organisms [6], [7].

The budding yeast *Saccharomyces cerevisiae* has only three PRMT genes, *HMT1*, *RMT2*, and *HSL7* [8]. *HMT1* (type I) is the major arginine methyltransferase and possesses similar functions of mammalian PRMT1. *HMT1* is not essential for cell growth in yeast. However, deletion of *HMT1* is synthetically lethal with mutations in the *NPL3* or *CBP80* genes [9]. *RMT2* is a type IV PRMT gene that is found in fungi and plants but not in protozoa and human [2]. The *HSL7* gene (type II) is orthologous to human *PRMT5* [8]. In Arabidopsis, many RNA binding or processing proteins are methylated by *AtPRMT5*. Mutations in the *AtPRMT5* gene affected RNA splicing in hundreds of genes involved in different biological processes and causes pleiotropic developmental defects, such as late flowering [10].

In *S. cerevisiae*, Hmt1 is a non-essential member of the heterogeneous nuclear ribonucleoproteins (hnRNPs) that are involved in mRNA biogenesis [9]. It confers SAM-dependent methylation to components of hnRNPs, which often contain C-terminal RGG-rich repeats as the sites of arginine methylation [11]. Hrp1, Nab2, and Npl3 are among the most studied hnRNPs that are methylated by Hmt1. Methylation by Hmt1 is important for their export from the nucleus [9], [11], [12]. Hrp1 is involved in the processing of 3′ ends of pre-mRNA, mRNA polyadenylation, and the nonsense mediated decay pathway [13], [14], [15]. The Nab2 protein is required for the export of poly(A) RNA and poly(A) tail length control [12], [16]. Npl3 has been implicated in transcription elongation and termination [17]. Methylation by Hmt1 in the nucleus and phosphorylation by the SR protein kinase Sky1 in the cytoplasm regulate the nucleo-cytoplasmic transport of Npl3 [18].

Orthologs of yeast PRMT genes are well conserved in filamentous plant pathogenic ascomycetes. However, none of them have been experimentally characterized for their biological functions in plant pathogenic ascomycetes. *F. graminearum* is a major causal agent of wheat and barley head blight or scab worldwide [19], [20]. Fusarium head blight (FHB) poses as a serious problem in wheat production by causing severe yield losses and contamination of infested kernels with harmful mycotoxins, including deoxynivalenol (DON) and zearalenone [19], [21]. Because of the importance of PRMT genes in eukaryotes [2], [5], in this study we identified and functionally characterized all of the four predicted PRMT genes in *F. graminearum*. Whereas deletion of the other three PRMT genes had no obvious phenotypes, the Δ*amt1* mutant was significantly reduced in virulence and DON production in infection assays with flowering wheat heads. Our results indicate that *AMT1*, like its ortholog *HMT1* in yeast, is the predominant arginine methyltransferase in *F. graminearum*. Although dispensable for sexual and asexual reproduction, *AMT1* is important for normal growth rate, stress responses, plant infection, and nucleo-cytoplasmic transport of FgHrp1.

## Results

### Identification of the *HMT1* ortholog, *AMT1*, in *F. graminearum*

The genome of *F. graminearum* contains four PRMT genes, FGSG_01134 (XP_381310), FGSG_10718 (XP_390894), FGSG_00501 (XP_380677), and FGSG_10756 (XP_390932) that are named *AMT1*-*AMT4* (for arginine methyltransferase genes) in this study. FGSG_01134 (*AMT1*) is orthologous to *HMT1*, which is the main arginine methyltransferase gene in *S. cerevisiae*. The 345-amino acid protein encoded by *HMT1* has a typical arginine methyltransferase domain. FGSG_10718 (*AMT2*) encodes a PRMT3-like protein that also shares significant homology with yeast *HMT1*. FGSG_00501 (*AMT3*) and FGSG_10756 (*AMT4*) are orthologous to yeast *RMT2* and *HSL7*, respectively (Figure S1). Unlike the budding yeast, filamentous ascomycetes such as *Magnaporthe oryzae* and *Aspergillus nidulans* (Figure S1) have four PRMT genes.

### Generation of *amt1* deletion mutants

**Figure 1:**
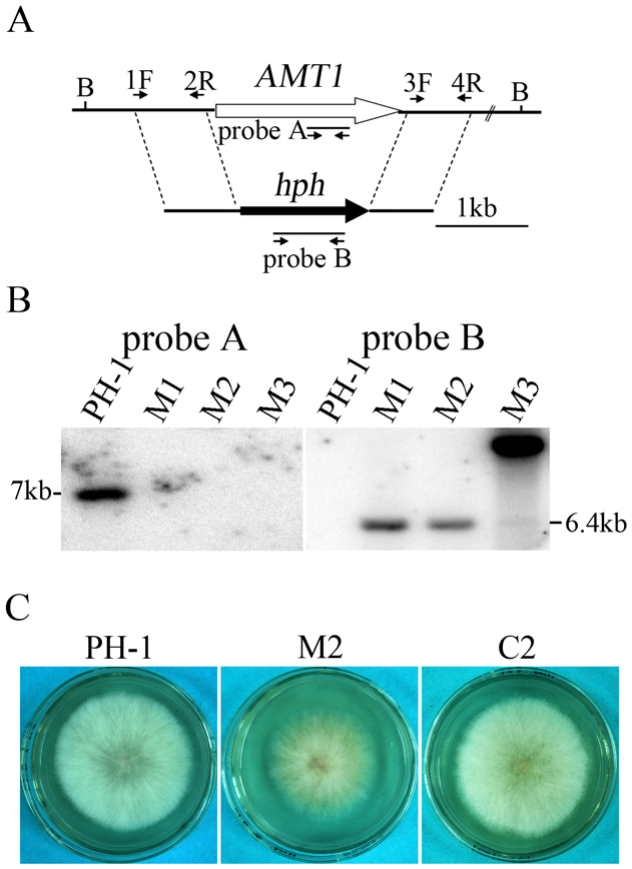
The *AMT1* gene replacement construct and deletion mutants. **A.** The *AMT1* locus and gene replacement construct. The *AMT1* and *hph* genes are marked with empty and black arrows, respectively. 1F, 2R, 3F, and 4R are primers used to amplify the flanking sequences. *Bam*HI (B). **B.** Southern blot analysis with the wild type (PH-1) and Δ*amt1* transformants (M1, M2, and M3). All DNA samples were digested with *Bam*HI. The blots were hybridized with probe A (left) amplified with primers AMT1/5F and AMT1/6R and probe B (right) amplified with H852 and H850. **C.** Colony morphology of the PH-1, Δ*amt1* mutant M2, and Δ*amt1/AMT1* transformant C2 cultures grown on CM. Photographs were taken after incubation for 3 days.

The *AMT1* gene replacement construct (Fig. 1A) was generated with the split-marker approach and transformed into the wild-type strain PH-1. Putative Δ*amt1* mutants were identified by PCR and confirmed by Southern blot analysis (Fig. 1B). In the wild type, a 7.0-kb *Bam*HI band was detected with an *AMT1* fragment amplified with primers AMT1/5F and AMT1/6R (Table S2) as the probe A (Fig. 1B). The same probe had no hybridization signal in transformants M1, M2, and M3 (Table 1). When probed with a fragment of the *hph* gene, PH-1 had no hybridization signals. Transformants M1 and M2 had a 6.4-kb band (Fig. 1B), which is similar to the expected size derived from the gene replacement event (Fig. 1A). Transformant M3 had a weak 6.4-kb band but a strong 10-kb band, suggesting that besides targeted homologous recombination, multiple copies of the *AMT1* gene replacement construct were integrated ectopically during transformation. Therefore, only transformants M1 and M2 were the expected *amt1* deletion mutants with no additional integration events. Mutants M1 and M2 had the same phenotype although only data with mutant M2 were described below.

**Table 1:** The wild-type and mutant strains of *Fusarium graminearum* used in this study.

Strains	Brief descriptions	Reference
PH-1	Wild-type	[55]
M1	Δ*amt1* mutant of PH-1	This study
M2	Δ*amt1* mutant of PH-1	This study
M3	Δ*amt1* mutant of PH-1	This study
C2	Δ*amt1*/*AMT1* transformant of M2	This study
Y5	Δ*amt1*/*AMT1-*GFP transformant of M2	This study
HP10	Transformant of PH-1 expressing *FgHRP1*-GFP	This study
HA11	Transformant of M2 expressing *FgHRP1*-GFP	This study
NP12	Transformant of PH-1 expressing *FgNAB2*-GFP	This study
NA14	Transformant of M2 expressing *FgNAB2*-GFP	This study
DM7	Δ*amt1* Δ*amt2* double mutant	This study
DM12	Δ*amt1* Δ*amt2* double mutant	This study
KS2	Δ*amt2* (FGSG_10718) deletion mutant of PH-1	This study
KT3	Δ*amt3* (FGSG_00501) deletion mutant of PH-1	This study
KF4	Δ*amt4* (FGSG_10756) deletion mutant of PH-1	This study

When assayed for growth on CM medium, the Δ*amt1* mutant produced less aerial hyphae than the wild type (Fig. 1C) and had approximately 24% reduction in growth rate (Table 2). It was also reduced in aerial hyphal growth and growth rate on PDA, 5×YEG, and YEPD plates (Figure S2). When the wild-type *AMT1* allele was transformed into the Δ*amt1* mutant, defects in hyphal growth and other phenotypes described below were rescued in the resulting Δ*amt1/AMT1* transformant C2 (Table 2). These results indicate that deletion of *AMT1* is directly responsible for the growth defects observed in the mutant and *AMT1* is important for normal vegetative growth in *F. graminearum*.

**Table 2:** Defects of the Δ*amt1* mutant in growth, conidiation, and plant infection.

	Growth rate (mm/d)^a^	Conidiation (10^6^/ml)	Disease Index^b^	DON (ppm)
PH-1 (WT)	14.0±0.0^A^ *	1.39±0.16^A^	13.8±3.8^A^	1589.6±359.4^A^
M2 (Δ*amt1*)	10.6±0.3^B^	1.45±0.25^A^	4.3±3.7^B^	397.0±188.7^B^
C2 (Δ*amt1*/*AMT1*)	14.3±0.2^A^	1.42±0.21^A^	14.0±3.4^A^	1782.8±279.6^A^

a Average growth rate/conidiation and standard error (mean ± standard error) were calculated from at least three independent measurements.

b Disease was rated by the number of symptomatic spikeletes 14 days after inoculation. Mean and standard error were calculated with results from three independent experiments. At least 10 wheat heads were examined in each repeat.

* Data from three replicates were analyzed with the protected Fisher's Least Significant Difference (LSD) test. The same letter indicated that there was no significant difference. Different letters were used to mark statistically significant difference (P = 0.05).

### *AMT1* is dispensable for sexual and asexual reproduction

The Δ*amt1* mutant was normal in sexual reproduction (Figure S3). On mating plates, abundant perithecia were formed by the Δ*amt1* mutant. Ascospore cirrhi were observed on top of mature perithecia 3 weeks after fertilization (Figure S3). The Δ*amt1* mutant also had no obvious defects in conidiation (Table 2) and conidium germination. The morphology of Δ*amt1* ascospores and conidia was normal. These data indicates that although the Δ*amt1* mutant was slightly reduced in vegetative growth, *AMT1* is dispensable for sexual and asexual reproduction.

### The Δ*amt1* mutant is significantly reduced in virulence

**Figure 2:**
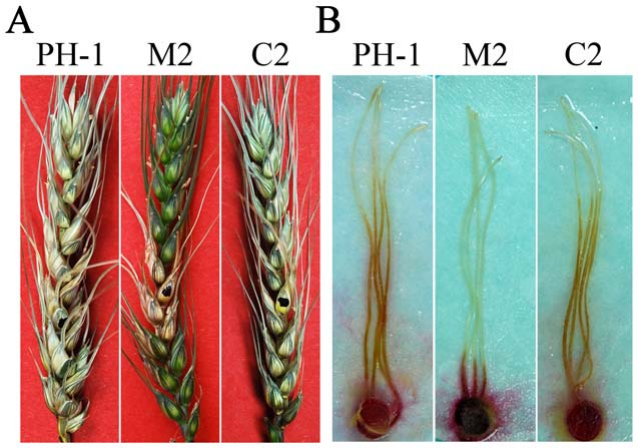
Infection assays with flowering wheat heads and corn silks. **A.** Flowering wheat heads were drop-inoculated with conidia from the wild-type (PH-1), Δ*amt1* mutant (M2), and Δ*amt1/AMT1* (C2) strains. Black dots mark the inoculated spikeletes. Photographs were taken 14 days post-inoculation (dpi). **B.** Corn silks were inoculated with blocks of cultures of PH-1, Δ*amt1* mutant M2, and Δ*amt1/AMT1* transformant C2. Photographs were taken 6 dpi.

In infection assays with flowering wheat heads, the Δ*amt1* mutant caused typical scab symptoms in the inoculated kernels and was able to spread to nearby spikeletes (Fig. 2A). However, it was significantly reduced in virulence compared to PH-1 (Fig. 2A). The average disease index, a measurement for virulence by counting diseased spikeletes per wheat head, of the Δ*amt1* mutant and PH-1 was 4.3 and 13.8, respectively (Table 2), indicating that the Δ*amt1* mutant was defective in disease spreading.

Corn also is a host to *F. graminearum*. In infection assays with corn silks, the Δ*amt1* mutant caused only limited discoloration near the inoculation sites. Under the same conditions, extensive discoloration was observed in corn silks inoculated with PH-1 (Fig. 2B), confirming that *AMT1* is important for virulence in *F. graminearum*.

**Figure 3:**
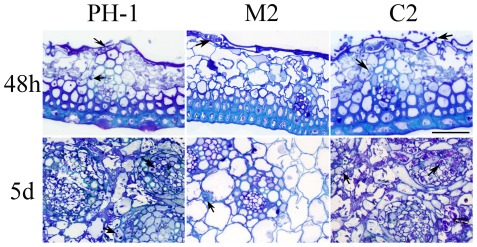
Colonization of flowering wheat heads. **A.** Colonization of glume tissues by the wild-type (PH-1), Δ*amt1* mutant (M2), and Δ*amt1/AMT1* (C2) strains 48 hpi. **B.** The rachises directly beneath the inoculated spikeletes were examined 120 hpi. Hyphae growth (marked with arrows) was abundant in plant tissues inoculated with the wild type and Δ*amt1/AMT1* strains but scarce in samples inoculated with Δ*amt1* mutant. Bar = 50 µm.

To characterize the defects of the Δ*amt1* mutant in plant infection, inoculated flowering wheat heads were sampled, fixed, and examined for hyphal growth. At 48 h post-inoculation (hpi), fungal growth was observed on the surface and inside glume tissues inoculated with the wild type (Fig. 3). In wheat heads inoculated with the Δ*amt1* mutant, fungal growth was abundant on the surface and rarely in glume tissues (Fig. 3). At 120 hpi, the wild type had colonized the vascular and other tissues of the rachis and produced abundant intracellular hyphae (Fig. 3). In contrast, fungal growth was limited or much sparse in the rachis near the spikeletes inoculated with the Δ*amt1* mutant (Fig. 3), indicating that *AMT1* is important for invasion and spreading in plant tissues. These observations are consistent with reduced virulence of the Δ*amt1* mutant.

### The Δ*amt1* mutant has increased sensitivity to oxidative and membrane stresses

**Figure 4:**
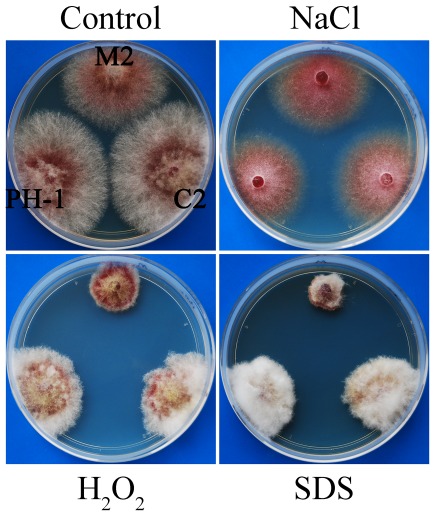
Assays for defects of the Δ*amt1* mutant to different stresses. Cultures of the wild-type (PH-1), Δ*amt1* mutant (M2), and Δ*amt1/AMT1* (C2) strains grown on regular PDA or PDA with 0.7 M NaCl, 0.05% H_2_O_2_, or 0.01% SDS. Photographs were taken after incubation at 25°C for 3 days.

To determine whether the Δ*amt1* mutant was defective in stress responses, we assayed its growth on PDA plates with 0.05% H_2_O_2_, 0.01%SDS, and 0.7 M NaCl. It appears that *AMT1* is dispensable for responses to hyperosmotic stress because the wild type and Δ*amt1* mutant stains had no obvious difference in vegetative growth on PDA plates with 0.7 M NaCl (Fig. 4). However, *AMT1* is likely involved in responses to oxidative and membrane stresses. In the presence of 0.05% H_2_O_2_ or 0.01%SDS, the Δ*amt1* mutant was more significantly reduced in growth rate than the wild type (Fig. 4).

### Subcellular localization of *AMT1*-GFP fusion

**Figure 5:**
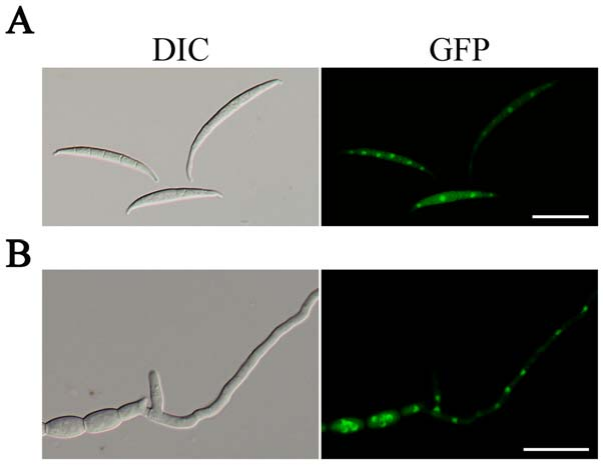
Subcellular localization of Amt1-GFP fusion proteins. Conidia (**A**) and 7 h germlings (**B**) of the Δ*amt1/AMT1*-GFP transformant (Y5) were examined by phase contrast (DIC) or epifluorescence (GFP) microscopy. GFP signals were present in both the cytoplasm and nucleus but were stronger in the nucleus. Bar = 50 µm.

To determine the expression and localization of *AMT1*, we generated an *AMT1*-GFP fusion construct and transformed it into the Δ*amt1* mutant. After screened by PCR and confirmed by Southern blot analysis, transformant Y5 (Table 1) was identified as one of the transformants expressing the *AMT1*-GFP construct under the control of its native promoter. Similar to the complemented strain C2, defects of the Δ*amt1* mutant were rescued in the Δ*amt1/AMT1*-GFP transformant. GFP signals were present in both cytoplasm and nuclei in conidia and 7 h germlings of transformant Y5 (Fig. 5). However, nuclei had stronger fluorescence signals than the cytoplasm, indicating that majority of Amt1-GFP proteins localized to the nucleus.

### Amt1 influences the nuclear transport of FgHrp1

**Figure 6:**
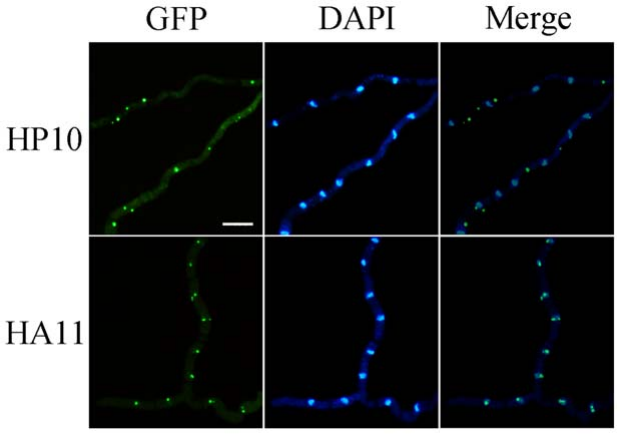
Amt1 regulates the nucleo-cytoplasmic transport of FgHrp1. Fresh conidia were harvested from transformants of PH-1 (HP10) and Δ*amt1* mutant M2 (HA11) expressing the *FgHRP1*-GFP fusion construct and incubated in liquid YEPD medium for 12 h. Germ tubes were then stained with DAPI and examined by fluorescence microscopy with the GFP- and DAPI-specific filters. FgHrp1-GFP fusion proteins mainly localized to the nucleus in the Δ*amt1* mutant but to the cytoplasm in the wild type. Bar = 20 µm.

In *S. cerevisiae*, Hmt1 is involved in the regulation of nucleo-cytoplasmic transport of hnRNP components, including Hrp1 and Nab2 [9]. Orthologs of *HRP1* and *NAB2* in *F. graminearum* are FGSG_13728.3 and FGSG_01282.3, respectively. We constructed the *FgHRP1*-GFP and *FgNAB2*-GFP fusion constructs and transformed them into the wild-type and Δ*amt1* mutant strains. In the resulting transformants expressing the *FgHRP1*-GFP construct, the subcellular localization of FgHrp1-GFP fusion proteins differed significantly between the wild-type and Δ*amt1* mutant (Fig. 6). In the Δ*amt1* mutant, GFP signals were detected mainly in the nucleus. Each nucleus had one or more dots of bright GFP signals that may correspond to hnRNP particles associated with FgHrp1. In the wild type, GFP signals were primarily observed in the cytoplasm, indicating that deletion of *AMT1* affected the nuclear export of FgHrp1 proteins. Fluorescent particles in the cytoplasm may represent protein complexes that are associated with FgHrp1 after its exit from the nucleus.

In contrast, FgNab2-GFP proteins were distributed mainly in the nucleus in both wild-type and Δ*amt1* mutant strains (Figure S4), indicating that *AMT1* is not important for the subcellular localization of FgNab2 in *F. graminearum*. Therefore, arginine methylation may play different roles in the nucleo-cytoplasmic transport of different hnRNP components in *F. graminearum* and *S. cerevisiae*.

### Functional characterization of the other three PRMT genes in *F. graminearum*

**Figure 7:**
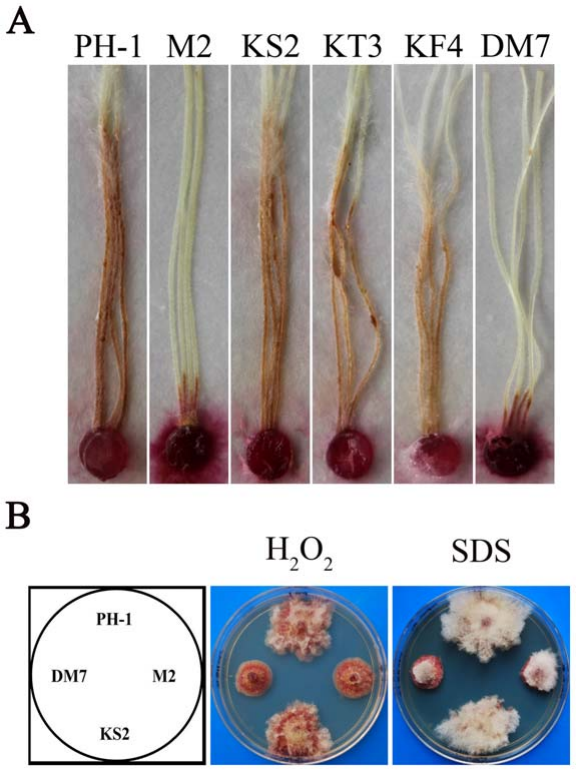
Infection assays and stress response tests with mutants deleted of other PRMT genes. **A**. Corn silks inoculated with the wild type (PH-1), M2 (Δ*amt1*), KS2 (Δ*amt2*), KT3 (Δ*amt3*), KF4 (Δ*amt4*), and DM7 (Δ*amt1* Δ*amt2*). **B**. Colonies of PH-1, DM7, M2, and KS2 grown on PDA plates with 0.05% H_2_O_2_ or 0.01% SDS.

To determine the functions of other three putative PRMT genes, the split-marker approach was used to generate the *AMT2* (FGSG_10718), *AMT3* (FGSG_00501), and *AMT4* (FGSG_10756) gene replacement constructs. The resulting PCR products were transformed into protoplasts of the wild-type strain PH-1. The Δ*amt2*, Δ*amt3*, and Δ*amt4* knockout mutants (Table 1) were identified by PCR and confirmed by Southern blot analyses. In comparison with the wild type, the Δ*amt2*, Δ*amt3*, and Δ*amt4* mutants had no obvious defects in vegetative growth, conidiation, and production of perithecia and ascospores (Figure S3; Figure S5). They also had similar growth rate with the wild type on PDA plates with 0.05% H_2_O_2_, 0.01% SDS, or 0.7 M NaCl, or 300 µg/ml Congo red (Figure S6). In infection assay with corn silks, the Δ*amt2* and Δ*amt3* mutants were as virulent as the wild type but the Δ*amt4* was slightly reduced in virulence (Fig. 7A; Table S1). These results indicate that *AMT2*, *AMT3*, and *AMT4* genes are dispensable for vegetative growth, asexual and sexual reproduction, and stress responses. While *AMT2* and *AMT3* were dispensable for plant infection, *AMT4* was required for full virulence.

### The Δ*amt1* Δ*amt2* double mutant has similar defects in plant infection with the Δ*amt1* mutant

The *AMT1* and *AMT2* genes are the only two predicted type I arginine methyltransferase genes in *F. graminearum*. They both share sequence similarity with yeast Hmt1 (Figure S1). To determine their functional relationship, we generated the Δ*amt1* Δ*amt2* double mutant by deletion of *AMT2* in the Δ*amt1* mutant M2. The resulting double mutant (Table 1), similar to the Δ*amt1* mutant, was normal in conidiation and sexual reproduction (Figure S3) but slightly reduced in vegetative growth (Figure S5). In plant infection assays, the Δ*amt1* Δ*amt2* had similar defects in virulence with the Δ*amt1* mutant (Fig. 7A). On PDA plates with 0.05% H_2_O_2_ or 0.01% SDS, the Δ*amt1* and Δ*amt1* Δ*amt2* mutants also had similar growth defects (Fig. 7B). Therefore, the Δ*amt1* and Δ*amt1* Δ*amt2* mutants had no significant differences in growth, stress responses, and virulence. These results suggest that *AMT1* and *AMT2* have no overlapping functions.

### Deletion of *AMT1* results in less than 2-fold changes in the expression of *AMT2*, *AMT3*, and *AMT4*

**Figure 8:**
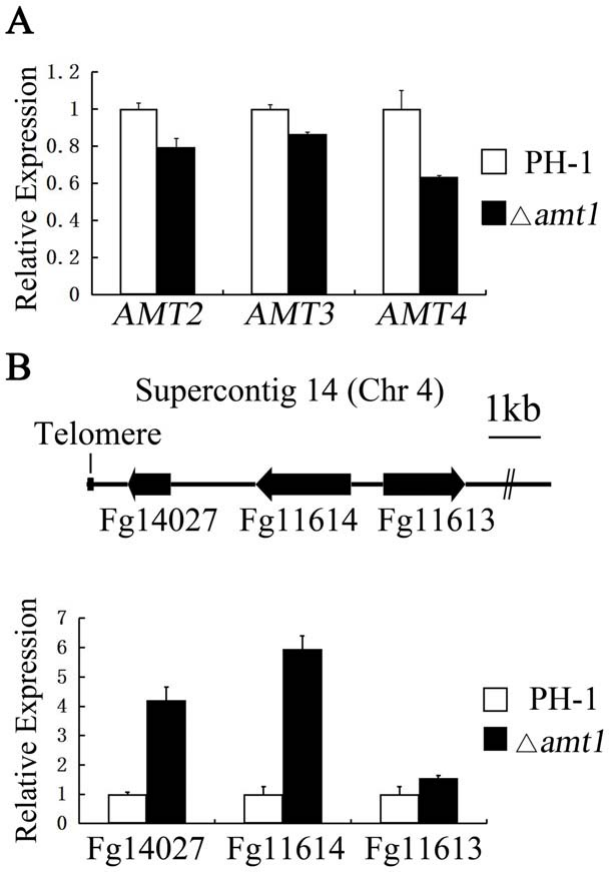
Assays for the effects of Δ*amt1* mutation on other PRMT genes and genes adjacent to the telomere. RNA samples were isolated from germlings of the wild-type (PH-1) and Δ*amt1* mutant strains grown in liquid YEPD for 6 h. The expression levels of (**A**) three other PRMT genes, *AMT2*, *AMT3*, and *AMT4*, and (**B**) three predicted genes located in the telomeric region of chromosome 4 (FGSG_14027, FGSG_11614, and FGSG_11613) were assayed by qRT-PCR.

RNA samples were isolated from vegetative hyphae of PH-1 and Δ*amt1* mutant grown in liquid CM for 6 h. In comparison with the wild type, the expression levels of *AMT2*, *AMT3*, and *AMT4* were reduced approximately 21%, 10%, and 44%, respectively, in the Δ*amt1* mutant (Fig. 8A). However, none of them had over 2-fold changes in the expression level between the wild type and Δ*amt1* mutant strains.

### Deletion of *AMT1* affects the expression of genes adjacent to the telomere

Because deletion of *HMT1* is known to affect the formation of silent chromatin [22], we assayed the expression of three genes, FGSG_14027, FGSG_11614, and FGSG_11613 that are within 10 kb from the telomeric repeat sequences (TTAGGG) on supercontig 14 (Chromosome 4, Fig. 8B). The current version of *F. graminearum* assembly contains no other telemetric repeat sequences. FGSG_14027 (795–1625) encodes a putative histone deacetylase gene orthologous to yeast *HOS4*. It is less than 1 kb away from the telomeric repeats. FGSG_11614 (3250–5086) and FGSG_11613 (5679–7261) encode hypothetical proteins that are conserved in filamentous ascomycetes but not in yeast. Whereas the expression level of FGSG_14027 and FGSG_11614 was increased over approximately 4- and 5-fold, respectively, expression of FGSG_11613 was slightly increased but not significantly in the Δ*amt1* mutant compared to the wild type (Fig. 8B). Since FGSG_11613 is more distal to the telomeric repeats than the other two genes, it is likely that silencing of genes adjacent to the telomere is affected by deletion of *AMT1*.

### The expression and activation of Mgv1, Gpmk1, and Fghog1 MAP kinases in the *amt1* mutant

**Figure 9:**
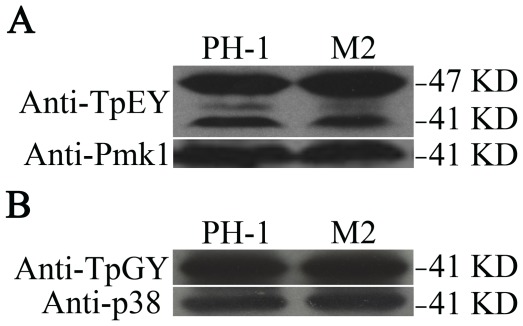
MAP kinase phosphorylation assays. Total proteins were isolated from the wild-type (PH-1) and *amt1* mutant (M2) strains. When detected with an anti-TpEY antibody, the phosphorylation levels of Mgv1 had no significant changes in the *amt1* mutant in comparison with the wild type. The phosphorylation of FgHog1 detected with an anti-TpGY antibody also appeared to be normal in the *amt1* mutant. Detection with a monoclonal anti-actin antibody showed equal amount of proteins.

Because PRMTs are known to affect signal transduction in mammalian cells [3], we assayed the phosphorylation of all three MAP kinases that have been characterized in *F. graminearum* and known to important for plant infection [23], [24], [25], [26], [27]. In comparison with the wild type strain, the *amt1* mutant was normal in the expression and phosphorylation levels of Mgv1 and Fghog1 (Fig. 9). For Gpmk1, the expression level was normal but the phosphorylation level was slightly but not significantly reduced (Fig. 9). These data indicate that Amt1 does not significantly affect the expression and activation of these MAP kinases in *F. graminearum*.

## Discussion

Methylation of the arginine residues by arginine methyltransferases plays important roles in various cellular processes in eukaryotic organisms such as nucleo-cytoplasmic transport and mRNA biogenesis [1], [3], [4]. The genome of *F. graminearum* contains four predicted arginine methyltransferase genes that belong to the type I, type II, and type VI of PRMTs [28], [29]. Phenotype analyses with targeted deletion mutants of these PRMT genes indicated that only the Δ*amt1* mutant had obvious defects in growth and plant infection. Mutants deleted of the other three PRMT genes had no significant phenotypes. Therefore, similar to its ortholog in yeast, *AMT1* must be the predominant arginine methyltransferase in *F. graminearum*. In *A. nidulans*, three *AMT1* genes, *rmtA*, *rmtB*, and *rmtC* that are orthologous to *AMT1*, *AMT2*, and *AMT4*, respectively, have been characterized. None of the *rmtA*, *rmtB*, and *rmtC* deletion mutants had obvious defects in vegetative growth, sexual, and asexual reproduction on normal growth conditions [30], suggesting that *A. nidulans* may lack a predominant PRMT gene.

*AMT2* encodes a predicted type I PRMT protein that shares significant sequence similarity to PRMT3 in human. Its orthologs are well conserved in filamentous fungi, including *M. oryzae*, *A. nidulans*, and *Neurospora crassa* and the fission yeast *Schizosaccharomyces pombe*. However, *S. cerevisiae* and *Candida albicans* lack a distinct ortholog of *AMT2*, suggesting that this gene may have been lost in some *Saccharomycetales* species during evolution. As the only other predicted type I PRMT gene in *F. graminearum*, *AMT2* shares limited homology with *AMT1* and yeast *HMT1*. Although Amt2 has a C2H2 zinc finger domain that is absent in Amt1, deletion of the *AMT2* gene had no obvious phenotypic changes. In *A. nidulans*, deletion of the *rmtB* gene also lacked any detectable phenotype [30]. To determine the relationship between *AMT1* and *AMT2*, we deleted the *AMT2* gene in the Δ*amt1* mutant. The Δ*amt1* mutant and the Δ*amt1* Δ*amt2* double mutant had no significant differences in the phenotypes assayed, including growth rate, sensitivities to oxidative stress, and virulence. Deletion of *AMT1* also had no significant impact on the expression level of *AMT2* (Fig. 8A). Therefore, it is unlikely for *AMT2* to have overlapping functions with *AMT1* in *F. graminearum*.

For the other two PRMT genes in *F. graminearum*, *AMT3* and *AMT4* are orthologous to the *RMT2* and *HSL7* genes in yeast, respectively. Rmt2 and its related PRMT genes are specific to fungi and plants [31]. In yeast, Rmt2 specifically methylates ribosomal protein Rpl12 (L12) on Arg67 [32]. The *rmt2* mutant is defective in δ N^G^ -methylarginine modifications but normal in growth and reproduction [33]. In *C. albicans*, the *rmt2*/*rmt2* mutant grew as robustly as the reconstituted or heterozygous strains in rich media but the level of δ N^G^-monomethylarginine is reduced [31]. However, no data on virulence of the mutant were presented. The genome of *A. nidulans* contains the ortholog of *AMT3* (Figure S1) but it has not been experimentally characterized for its biological function [30].

In *S. cerevisiae*, Hsl7 is required along with Hsl1 kinase for bud neck recruitment, phosphorylation, and degradation of Swe1 [34]. The Δ*hsl7* mutant produces elongated, anucleate buds and has increased sensitivity to Calcofluor and CaCl_2_ [34]. In *F. graminearum*, the *AMT4* deletion mutant had no obvious defects but the *rmtC* mutant of *A. nidulans* had increased sensitivity to oxidative stress and elevated temperatures [30]. In *U. maydis*, the Hsl7 ortholog was identified as a Smu1 PAK kinase interacting protein. It regulates cell length and the filamentous response to solid SLAD (synthetic low ammonia plus 2% dextrose) but is dispensable for plant infection. Although the *amt3* and *amt4* mutants of *F. graminearum* had no obvious defects in phenotypes assayed in this study, *AMT3* and *AMT4* genes are well conserved in filamentous fungi [35]. It is likely that they are functional in some biological processes that remain to be characterized in *F. graminearum*.

In *S. cerevisiae*, Hmt1 is a non-essential component of the hnRNP complex [9]. Hmt1 affects the nucleo-cytoplasmic transport of other hnRNP components that are important for mRNA biogenesis. In *F. graminearum*, the Δ*amt1* mutant was reduced approximately 24% in vegetative growth but normal in conidiation and ascospore production. If it also is a component of hnRNP in *F. graminearum*, Amt1 may be dispensable for mRNA processing of genes that are important for sexual reproduction and conidiation. In Arabidopsis, the *AtPRMT5* gene only affects RNA splicing in a subset of genes [10]. It is likely that only subsets of genes important for vegetative growth and plant infection (infectious growth) are affected in the Δ*amt1* mutant in *F. graminearum*. *AMT1* appears to play no or only minor roles in genes involved in sexual and asexual reproduction.

In infection assays with flowering wheat heads and corn silks, the Δ*amt1* mutant was significantly reduced in virulence. Although *AMT1* orthologs are well conserved, none of them have been characterized in plant pathogenic ascomycetes. In the human pathogen *Candida albicans*, deletion of *CaHMT1* affects the expression and localization of *NPL3* [31]. However, the function of *CaHMT1* in virulence has not been reported. One common stress faced by hyphae of necrotrophic fungi *in planta* is reactive oxygen species (ROS) generated during oxidative burst [36], [37]. The Δ*amt1* mutant, similar to the *rmtA* mutant of *A. nidulans* [30], had increased sensitivity to H_2_O_2_. It also had a slightly reduced growth rate and increased sensitivity to membrane stress. All of these defects may contribute to the defects of the Δ*amt1* mutant in plant infection. In addition, in diseased wheat kernels, the Δ*amt1* mutant was reduced in the production of DON, which is a well-characterized virulence factor in *F. graminearum* [38], [39]. However, reduced DON production in infested plant tissues may be related to reduced fungal biomass of the Δ*amt1* mutant.

In *S. cerevisiae*, two of the well-characterized hnRNP components are Hrp1 and Nab2 [12], [13]. *HRP1* is an essential gene that encodes a RRM-containing protein required for the cleavage and polyadenylation of pre-mRNA at the 3′-ends [13]. Nab2 is a nuclear polyA RNA-binding protein required for nuclear mRNA export and poly(A) tail length control. Methylation by Hmt1 regulates the shuttle of Hrp1 and Nab2 between the nucleus and cytoplasm. Hrp1 and Nab2 fail to exit the nucleus in cells lacking Hmt1 [11], [13]. In *F. graminearum*, FgHrp1-GFP fusion proteins mainly localized to the cytoplasm in the wild-type strain. In the Δ*amt1* mutant, FgHrp1-GFP proteins were accumulated in the nucleus (Fig. 5), suggesting that Amt1 is required for the nucleo-cytoplasm transport of FgHrp1. However, in transformants expressing the *FgNAB2*-GFP fusion construct, GFP signals mainly localized to the nucleus in both the wild type and Δ*amt1* mutant. The localization and nucleo-cytoplamic transport of FgNab2 appears to be independent of Amt1. These results indicate that methylation by this PRMT and nucleo-cytoplasmic transport of hnRNP components may be different between *S. cerevisiae* and *F. graminearum*.

## Materials and Methods

### Strains and culture conditions

The wild-type strain PH-1 and all the transformants of *F. graminearum* generated in this study were routinely cultured on PDA agar plates [24]. Growth rate and conidiation were assayed as described [40], [41]. DNA and RNA were extracted from vegetative hyphae harvested from liquid YEPD (1% yeast extract, 2% peptone, 2% glucose). Sexual reproduction, and protoplast preparation, and PEG-mediated transformation were performed as described [24]. Hygromycin B (Calbiochem, La Jolla, CA) and geneticin (Sigma, St. Louis, MO) were added to the final concentration of 300 and 350 µg/ml, respectively, to the TB3 medium for transformant selection. To test sensitivity against various stresses, vegetative growth was assayed on PDA plates with 0.05% H_2_O_2_ (v/v), 0.01% SDS (w/v), or 0.7 M NaCl as described [25], [42].

### Generation of Δ*amt1*, Δ*amt2*, Δ*amt3*, Δamt4, and Δ*amt1* Δ*amt2* mutants

All the mutants were generated with the split-marker approach [43]. For *AMT1*, the 0.83-kb upstream and 0.65-kb downstream flanking sequences were amplified with primer pairs AMT1/1F- 2R and AMT1/3F-4R, respectively (Fig. 1A and Table S2). The resulting PCR products were connected to the hygromycin phosphotransferase (*hph*) fragments amplified with primers HY/R-YG/F and HYG/F-HYG/R by overlapping PCR and transformed into protoplasts of PH-1 as described [40], [44]. Hygromycin-resistant transformants were screened for Δ*amt1* mutants by PCR with primer pairs AMT1F5-R6, AMT1F7-H855R, and H856F-AMT1R8 (Table S2). Putative Δ*amt1* mutants were then analyzed by Southern blot hybridizations to confirm the gene replacement event. The same approach was used to generate the Δ*amt2*, Δ*amt3*, and Δ*amt4* mutants. To generate the Δ*amt1* Δ*amt2* double mutant, the *AMT2* gene replacement construct generated with the neomycin resistance gene (*NEO^R^*) was transformed into the Δ*amt1* mutant M2.

### Complementation of the Δ*amt1* mutant

A fragment containing the entire *AMT1* gene and its promoter and terminator sequences was amplified with primers AMT1-CM/F and AMT1-CM/R (Table S2), digested with *Pst*I and *Bam*HI, and cloned between the *Pst*I and *Bam*HI sites of the *NEO^R^* vector pHZ100 [45]. The resulting construct, pAMT1, was transformed into protoplasts of the Δ*amt1* mutant M2. The Δ*amt1*/*AMT1* transformants were confirmed by PCR and Southern blot analyses.

### Generation of *AMT1*-GFP, *HRP1*-GFP, and *NAB2*-GFP fusion constructs

To generate the *AMT1*-GFP fusion, PCR products amplified with primers AMT1-YA/F and AMT1-YA/R (Table S2) were cloned into pFL2 by the yeast gap repair approach [46], [47]. The same approach was used to generating the *HRP1*-GFP and *NAB2*-GFP fusion constructs. All GFP fusion constructs were verified by sequencing analysis and transformed into protoplasts of PH-1 or the Δ*amt1* mutant M2. G418-resistant transformants harboring the transforming *AMT1*-GFP, *HRP1*-GFP, or *NAB2*-GFP construct were identified by PCR and confirmed by the presence of GFP signals.

### Infection and DON production assays

For infection assays with flowering wheat heads of cultivars XiaoYan 22 or Norm, conidia were harvested from 5-day-old CMC cultures and re-suspended in sterile distilled water to 2.0×10^5^ conidia/ml. The fifth spikelet from the base of the spike was inoculated with 10 µl of the conidial suspension as described [48]. Inoculated wheat heads were capped with a plastic bag to keep humidity for 48 h. After removing the bags, wheat plants were cultured for another 12 days before examination for symptomatic spikeletes. Infested kernels were harvested and assayed for DON production as described [45]. For microscopic examinations, glumes and rachises were collected from inoculated spikeletes and embedded in Spurr resins [49]. Thick sections (1 µm) were collected and placed on glass slides. After staining with aqueous 0.5% (w/v) toluidine blue, sections were examined and photographed with an Olympus BX-51 microscope (Olympus Corporation, Japan). Infection assays with corn silks of cultivar Pioneer 2375 were conducted as described [50].

### qRT-PCR analysis

RNA samples were isolated from 6 h germlings grown in liquid YEPD medium with the TRIzol reagent (Invitrogen, Carlsbad, CA). First-strand cDNA was synthesized with the Fermentas 1^st^ cDNA synthesis kit (Hanover, MD). All qRT-PCR reactions were performed with the Bio-Rad C1000 qRT-PCR machine. Primers used for qRT-PCR analysis were listed in Table S2. Relative expression levels of each gene were calculated by the 2^−ΔΔCt^ method [51] with the *F. graminearum* GAPDH gene [52] as the endogenous reference. Data from three biological replicates were used to calculate the mean and standard deviation.

### Western blot analysis

Total proteins were isolated from 24 h germlings grown in CM, separated on a 12.5% SDS-PAGE, and transferred to nitrocellulose membranes for western blot analysis as described [47], [53]. TEY-phosphorylation of Mgv1 and Gpmk1 and TGY- phosphorylation of FgHog1 were detected with the PhophoPlus p44/42 and p38 MAP kinase antibody kits (Cell Signaling Technology, Danvers, MA) following the manufacturer's instructions [54].

## Supporting Information

Figure S1 **Phylogenetic analysis of fungal PRMTs.** The amino acid sequences encoded by PRMT genes from *Fusarium graminearum*, *Candida albicans*, *Saccharomyces cerevisiae*, *Schizosaccharomyces pombe*, *Magnaporthe oryzae*, *Neurospora crassa*, *Aspergillus nidulans*, and *Homo sapiens* were analyzed by the DNAman5.0 program to create the dendrogram. The branch length is proportional to the mean number of differences per residue along each branch. All of the filamentous ascomycetes analyzed have four PRMT genes. Whereas three of them are orthologous to human *PRMT1*, *PRMT3*, and *PRMT5*, the fourth one is specific to fungi and plants. Scale bar is equal to 5% sequence divergence.
(TIF)

Figure S2 **Cultures of the wild type and Δ** ***amt1*** **mutant M2 grown on PDA, 5×YEG, and YEPD plates.**
(TIF)

Figure S3 **Perithecia and cirrhi produced by the wild-type strain (PH-1) and the Δ** ***amt1*** **(M2), Δ** ***amt2*** **(KS2), Δ** ***amt3*** **(KT3), Δ** ***amt4*** **(KF4), and Δ** ***amt1*** **Δ** ***amt2*** **(DM7) mutants.** Photographs were taken 14 days after fertilization.
(TIF)

Figure S4 **Deletion of AMT1 had no effects on the nucleo-cytoplasmic transport of FgNab2.** In both transformants of PH-1 (NP12) and Δ*amt1* (NA14) mutant expressing the *FgNAB2*-GFP fusion construct, GFP signals mainly localized to the nucleus. Bar = 20 µm.
(TIF)

Figure S5 **Three-day old PDA cultures of the wild-type strain (PH-1) and the Δ** ***amt2*** **(KS2), Δ** ***amt3*** **(KT3), Δ** ***amt4*** **(KF4), and Δ** ***amt1*** **Δ** ***amt2*** **(DM7) mutants.**
(TIF)

Figure S6 **Assays for defects in stress responses.** Cultures of the wild type (PH-1) and the Δ*amt2* (KS2), Δ*amt3* (KT3), and Δ*amt4* (KF4) mutants on PDA without or with 0.7 M NaCl, 300 µg/ml Congo red, 0.05% H_2_O_2_, or 0.01% SDS. Photographs were taken after incubation at 25°C for 3–5 days as labeled.
(TIF)

Table S1 **Disease index of AMTs mutants in the wheat head infection.**
(DOC)

Table S2 **PCR primers used in this study.**
(DOC)

## References

[1] Boisvert F, Chenard CA, Richard S (2005). Protein interfaces in signaling regulated by arginine methylation..

[2] Bachand F (2007). Protein arginine methyltransferases: From unicellular eukaryotes to humans.. Eukaryot Cell.

[3] Bedford MT, Richard S (2005). Arginine methylation: An emerging regulator of protein function.. Mol Cell.

[4] Yu MC, Bachand F, McBride AE, Komili S, Casolari JM (2004). Arginine methyltransferase affects interactions and recruitment of mRNA processing and export factors.. Genes Dev.

[5] Krause CD, Yang ZH, Kim YS, Lee JH, Cook JR (2007). Protein arginine methyltransferases: Evolution and assessment of their pharmacological and therapeutic potential.. Pharmacol Therapeut.

[6] Lee J, Sayegh J, Daniel J, Clarke S, Bedford MT (2005). PRMT8, a new membrane-bound tissue-specific member of the protein arginine methyltransferase family.. J Biol Chem.

[7] Scott HS, Antonarakis SE, Lalioti MD, Rossier C, Silver PA (1998). Identification and characterization of two putative human arginine methyltransferases (HRMT1L1 and HRMT1L2).. Genomics.

[8] Sayegh J, Clarke SG (2008). Hsl7 is a substrate-specific type II protein arginine methyltransferase in yeast.. Biochem Biophy Res Comm.

[9] McBride AE, Weiss VH, Kim HK, Hogle JM, Silver PA (2000). Analysis of the yeast arginine methyltransferase Hmt1p/Rmt1p and its in vivo function – Cofactor binding and substrate interactions.. J Biol Chem.

[10] Pei YX, Niu LF, Lu FL, Liu CY, Zhai JX (2007). Mutations in the type II protein arginine methyltransferase *AtPRMT5* result in pleiotropic developmental defects in Arabidopsis.. Plant Physiol.

[11] Shen EC, Henry MF, Weiss VH, Valentini SR, Silver PA (1998). Arginine methylation facilitates the nuclear export of hnRNP proteins.. Genes Dev.

[12] Green DM, Marfatia KA, Crafton EB, Zhang X, Cheng XD (2002). Nab2p is required for poly(A) RNA export in *Saccharomyces cerevisiae* and is regulated by arginine methylation via Hmt1p.. Jo Biol Chem.

[13] Kessler MM, Henry MF, Shen E, Zhao J, Gross S (1997). Hrp1, a sequence-specific RNA-binding protein that shuttles between the nucleus and the cytoplasm, is required for mRNA 3′-end formation in yeast.. Genes Dev.

[14] Gonzalez CI, Ruiz-Echevarria MJ, Vasudevan S, Henry MF, Peltz SW (2000). The yeast hnRNP-like protein Hrp1/Nab4 marks a transcript for nonsense-mediated mRNA decay.. Mol Cell.

[15] Gross S, Moore CL (2001). Rna15 interaction with the A-rich yeast polyadenylation signal is an essential step in mRNA 3 ′-end formation.. Mol Cell Biol.

[16] Hector RE, Nykamp KR, Dheur S, Anderson JT, Non PJ (2002). Dual requirement for yeast hnRNP Nab2p in mRNA poly(A) tail length control and nuclear export.. EMBO J.

[17] Wong CM, Tang HMV, Kong KYE, Wong GWO, Qiu HF (2010). Yeast arginine methyltransferase Hmt1p regulates transcription elongation and termination by methylating Npl3p.. Nucl Acids Res.

[18] Yun CY, Fu XD (2000). Conserved SR protein kinase functions in nuclear import and its action is counteracted by arginine methylation in *Saccharomyces cerevisiae*.. J Cell Biol.

[19] Goswami RS, Kistler HC (2004). Heading for disaster: *Fusarium graminearum* on cereal crops.. Mol Plant Pathol.

[20] McMullen M, Jones R, Gallenberg D (1997). Scab of wheat and barley: A re-emerging disease of devastating impact.. Plant Dis.

[21] Seong K, Li L, Hou ZM, Tracy M, Kistler HC (2006). Cryptic promoter activity in the coding region of the HMG-CoA rediactase gene in *Fusarium graminearum*.. Fungal Genet Biol.

[22] Yu MC, Lamming DW, Eskin JA, Sinclair DA, Silver PA (2006). The role of protein arginine methylation in the formation of silent chromatin.. Genes Dev.

[23] Ochiai N, Tokai T, Nishiuchi T, Takahashi-Ando N, Fujimura M (2007). Involvement of the osmosensor histidine kinase and osmotic stress-activated protein kinases in the regulation of secondary metabolism in *Fusarium graminearum*.. Biochem Biophy Res Comm.

[24] Hou ZM, Xue CY, Peng YL, Katan T, Kistler HC (2002). A mitogen-activated protein kinase gene (*MGV1*) in *Fusarium graminearum* is required for female fertility, heterokaryon formation, and plant infection.. Mol Plant-Microbe Interact.

[25] Wang C, Zhang S, Hou R, Zhao Z, Zheng Q (2011). Functional analysis of the kinome of the wheat scab fungus *Fusarium graminearum*.. PLoS pathogens.

[26] Jenczmionka NJ, Maier FJ, Losch AP, Schafer W (2003). Mating, conidiation and pathogenicity of *Fusarium graminearum*, the main causal agent of the head-blight disease of wheat, are regulated by the MAP kinase *gpmk1*.. Curr Genet.

[27] Urban M, Mott E, Farley T, Hammond-Kosack K (2003). The *Fusarium graminearum MAP1* gene is essential for pathogenicity and development of perithecia.. Mol Plant Pathol.

[28] Cimato TR, Tang J, Xu Y, Guarnaccia C, Herschman HR (2002). Nerve growth factor-mediated increases in protein methylation occur predominantly at type I arginine methylation sites and involve protein arginine methyltransferase 1.. J Neurosci Res.

[29] Zobel-Thropp P, Gary JD, Clarke S (1998). delta-N-methylarginine is a novel posttranslational modification of arginine residues in yeast proteins.. J Biol Chem.

[30] Bauer I, Graessle S, Loidl P, Hohenstein K, Brosch G (2010). Novel insights into the functional role of three protein arginine methyltransferases in *Aspergillus nidulans*.. Fungal Genet Biol.

[31] McBride AE, Zurita-Lopez C, Regis A, Blum E, Conboy A (2007). Protein arginine methylation in *Candida albicans*: role in nuclear transport.. Eukaryot Cell.

[32] Chern MK, Chang KN, Liu LF, Tam TCS, Liu YC (2002). Yeast ribosomal protein L12 is a substrate of protein-arginine methyltransferase 2.. J Biol Chem.

[33] Niewmierzycka A, Clarke S (1999). S-adenosylmethionine-dependent methylation in *Saccharomyces cerevisiae* – Identification of a novel protein arginine methyltransferase.. J Biol Chem.

[34] Kucharczyk R, Gromadka R, Migdalski A, Slonimski PP, Rytka J (1999). Disruption of six novel yeast genes located on chromosome II reveals one gene essential for vegetative growth and two required for sporulation and conferring hypersensitivity to various chemicals.. Yeast.

[35] Ben Lovely C, Aulakh KB, Perlin MH (2011). Role of Hsl7 in morphology and pathogenicity and its interaction with other signaling components in the plant pathogen *Ustilago maydis*.. Eukaryot Cell.

[36] Bolwell GP, Bindschedler LV, Blee KA, Butt VS, Davies DR (2002). The apoplastic oxidative burst in response to biotic stress in plants: a three-component system.. J Exp Bot.

[37] Torres MA, Jones JDG, Dangl JL (2006). Reactive oxygen species signaling in response to pathogens.. Plant Physiol.

[38] Proctor RH, Hohn TM, McCormick SP (1995). Reduced virulence of *Gibberella zeae* caused by disruption of a trichothecene toxin biosynthetic gene.. Mol Plant-Microbe Interact.

[39] Harris LJ, Desjardins AE, Plattner RD, Nicholson P, Butler G (1999). Possible role of trichothecene mycotoxins in virulence of *Fusarium graminearum* on maize.. Plant Dis.

[40] Zhou XY, Heyer C, Choi YE, Mehrabi R, Xu JR (2010). The *CID1* cyclin C-like gene is important for plant infection in *Fusarium graminearum*.. Fungal Genet Biol.

[41] Ding SL, Mehrabi R, Koten C, Kang ZS, Wei YD (2009). Transducin beta-like gene *FTL1* is essential for pathogenesis in *Fusarium graminearum*.. Eukaryot Cell.

[42] Li Y, Wang C, Liu W, Wang G, Kang Z (2011). The HDF1histone deacetylase gene is important for conidiation, sexual reproduction, and pathogenesis in *Fusarium graminearum*.. Mol Plant-Microbe Interact.

[43] Catlett NL, Lee B, Yoder OC, Turgeon BG (2003). Split-marker recombination for efficient targeted deletion of fungal genes.. Fungal Genet Newsl.

[44] Wang Y, Liu W, Hou Z, Wang C, Zhou X (2011). A novel transcriptional factor important for pathogenesis and ascosporogenesis in *Fusarium graminearum*.. Mol Plant-Microbe Interact.

[45] Bluhm BH, Zhao X, Flaherty JE, Xu JR, Dunkle LD (2007). RAS2 regulates growth and pathogenesis in Fusarium graminearum.. Molecular Plant-Microbe Interactions.

[46] Zhou X, Xu JR, Xu JR, Bluhm B (2011). Efficient approaches for generating GFP fusion and epitope-tagging constructs in filamentous fungi.. Fungal Genomics: Methods and Protocols.

[47] Bruno KS, Tenjo F, Li L, Hamer JE, Xu JR (2004). Cellular localization and role of kinase activity of *PMK1* in *Magnaporthe grisea*.. Eukaryot Cell.

[48] Gale LR, Chen LF, Hernick CA, Takamura K, Kistler HC (2002). Population analysis of *Fusarium graminearum* from wheat fields in eastern China.. Phytopathology.

[49] Kang ZS, Buchenauer H, Huang LL, Han QM, Zhang HC (2008). Cytological and immunocytochemical studies on responses of wheat spikes of the resistant Chinese cv. Sumai 3 and the susceptible cv. Xiaoyan 22 to infection by *Fusarium graminearum*.. Euro J Plant Pathol.

[50] Seong K, Hou ZM, Tracy M, Kistler HC, Xu JR (2005). Random insertional mutagenesis identifies genes associated with virulence in the wheat scab fungus *Fusarium graminearum*.. Phytopathology.

[51] Livak KJ, Schmittgen TD (2001). Analysis of relative gene expression data using real-time quantitative PCR and the 2(T)(-Delta Delta C) method.. Methods.

[52] Pandolfi V, Jorge EC, Melo CMR, Albuquerque ACS, Carrer H (2010). Gene expression profile of the plant pathogen *Fusarium graminearum* under the antagonistic effect of *Pantoea agglomerans*.. Genet Mol Res.

[53] Ding S, Liu W, LLiuk A, Ribot C, Vallet J (2010). The Tig1 HDAC complex regulates infectious growth in the rice blast fungus *Magnaporthe oryzae*.. Plant Cell.

[54] Liu W, Zhou X, Li G, Li L, Kong L (2011). Multiple plant surface signals are sensed by different mechanisms in the rice blast fungus for appressorium formation.. PLoS Pathogens.

[55] Cuomo CA, Gueldener U, Xu JR, Trail F, Turgeon BG (2007). The *Fusarium graminearum* genome reveals a link between localized polymorphism and pathogen specialization.. Science.

