# Supplementary figures and images for: The AMT1 Arginine Methyltransferase Gene Is Important for Plant Infection and Normal Hyphal Growth in Fusarium graminearum
Source: PLoS One. 2012 May 31;7(5):e38324. doi: 10.1371/journal.pone.0038324 (PMC3365026; doi:10.1371/journal.pone.0038324)

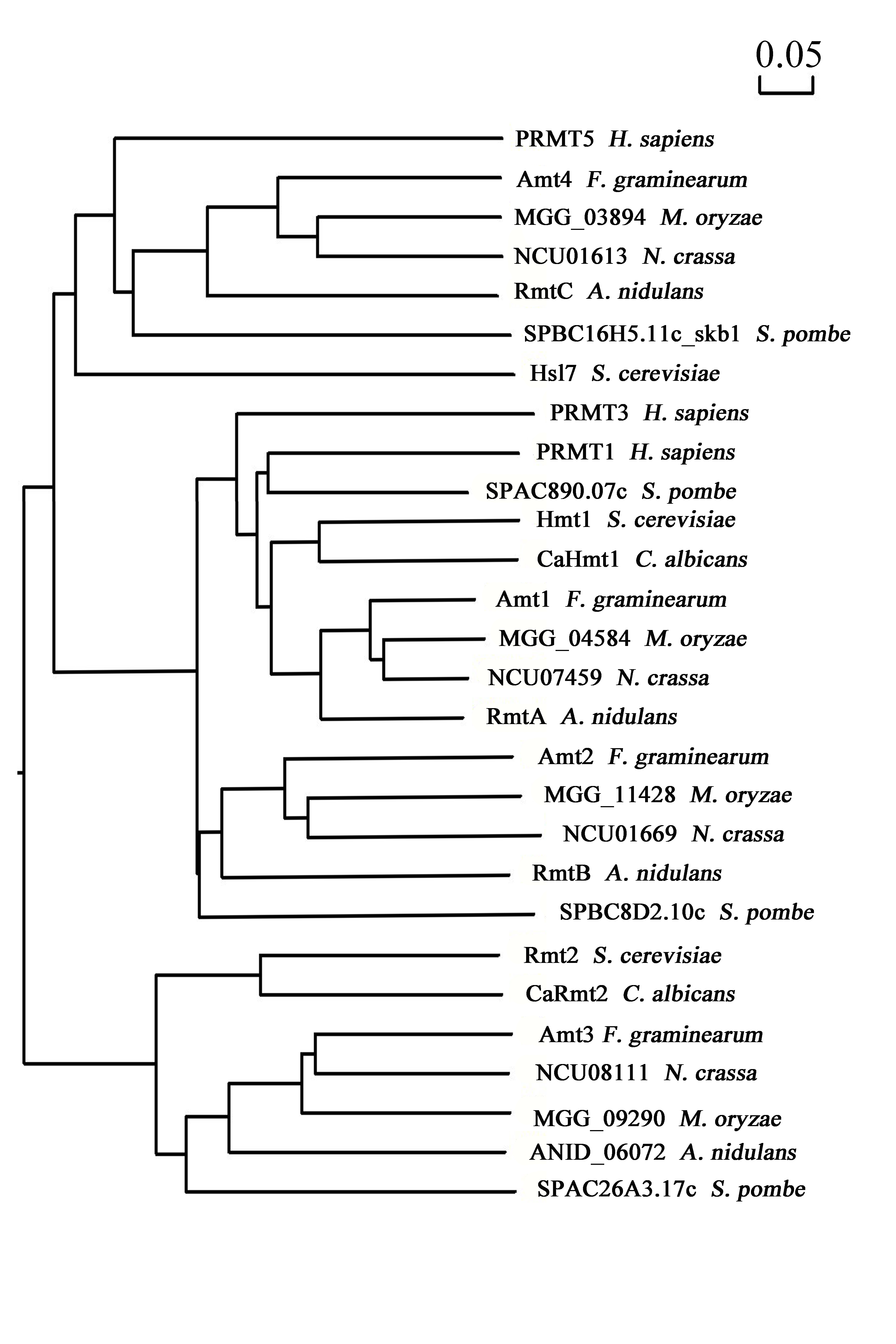

Supplement: Figure S1 — Phylogenetic analysis of fungal PRMTs. The amino acid sequences encoded by PRMT genes from Fusarium graminearum, Candida albicans, Saccharomyces cerevisiae, Schizosaccharomyces pombe, Magnaporthe oryzae, Neurospora crassa, Aspergillus nidulans, and Homo sapiens were analyzed by the DNAman5.0 program to create the dendrogram. The branch length is proportional to the mean number of differences per residue along each branch. All of the filamentous ascomycetes analyzed have four PRMT genes. Whereas three of them are orthologous to human PRMT1, PRMT3, and PRMT5, the fourth one is specific to fungi and plants. Scale bar is equal to 5% sequence divergence. (TIF) [file pone.0038324.s001.tif]

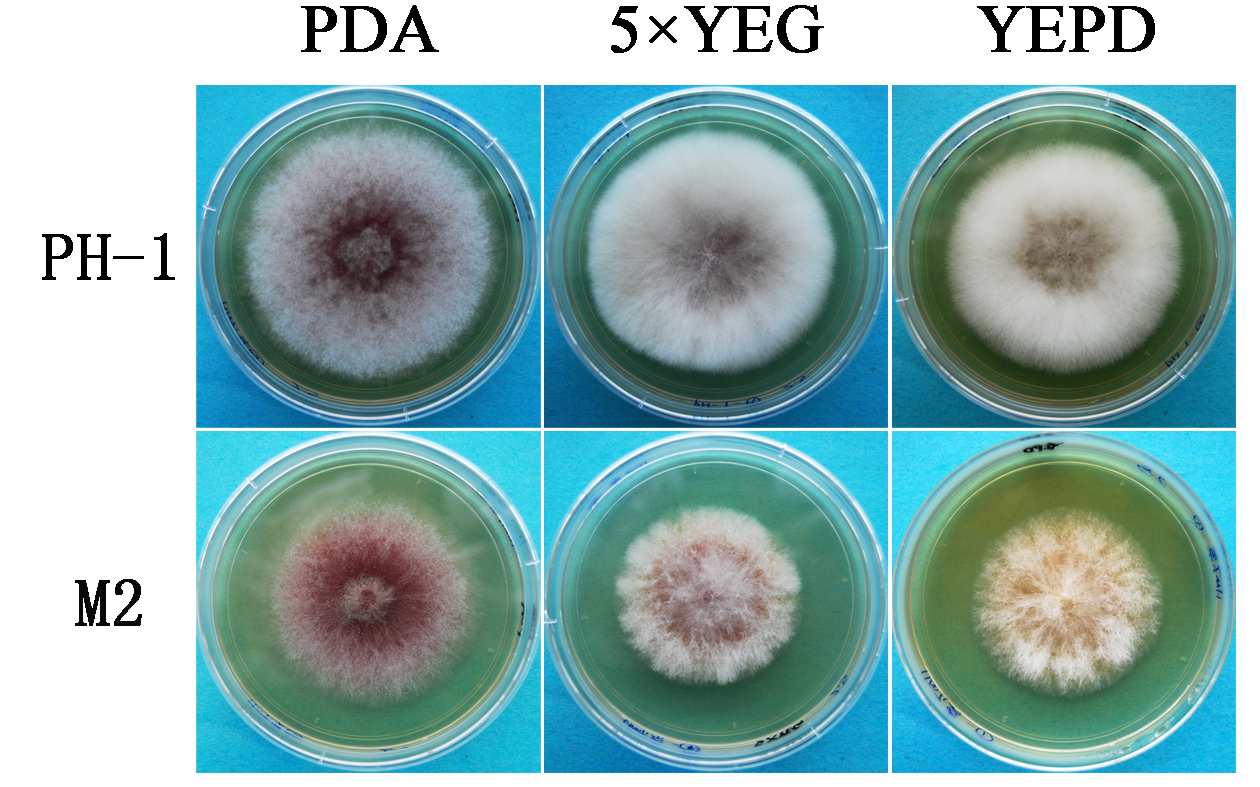

Supplement: Figure S2 — Cultures of the wild type and Δ amt1 mutant M2 grown on PDA, 5×YEG, and YEPD plates. (TIF) [file pone.0038324.s002.tif]

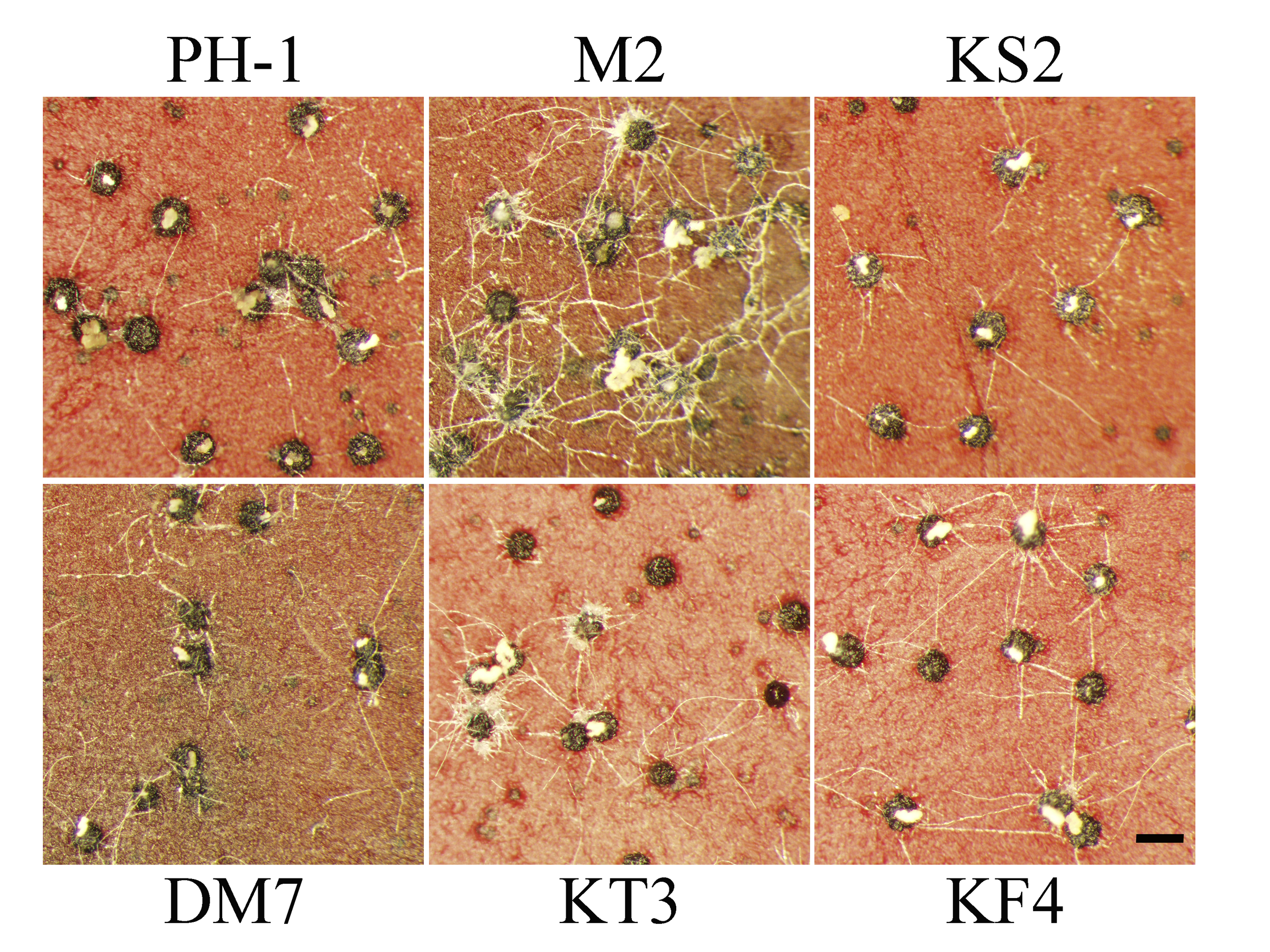

Supplement: Figure S3 — Perithecia and cirrhi produced by the wild-type strain (PH-1) and the Δ amt1 (M2), Δ amt2 (KS2), Δ amt3 (KT3), Δ amt4 (KF4), and Δ amt1 Δ amt2 (DM7) mutants. Photographs were taken 14 days after fertilization. (TIF) [file pone.0038324.s003.tif]

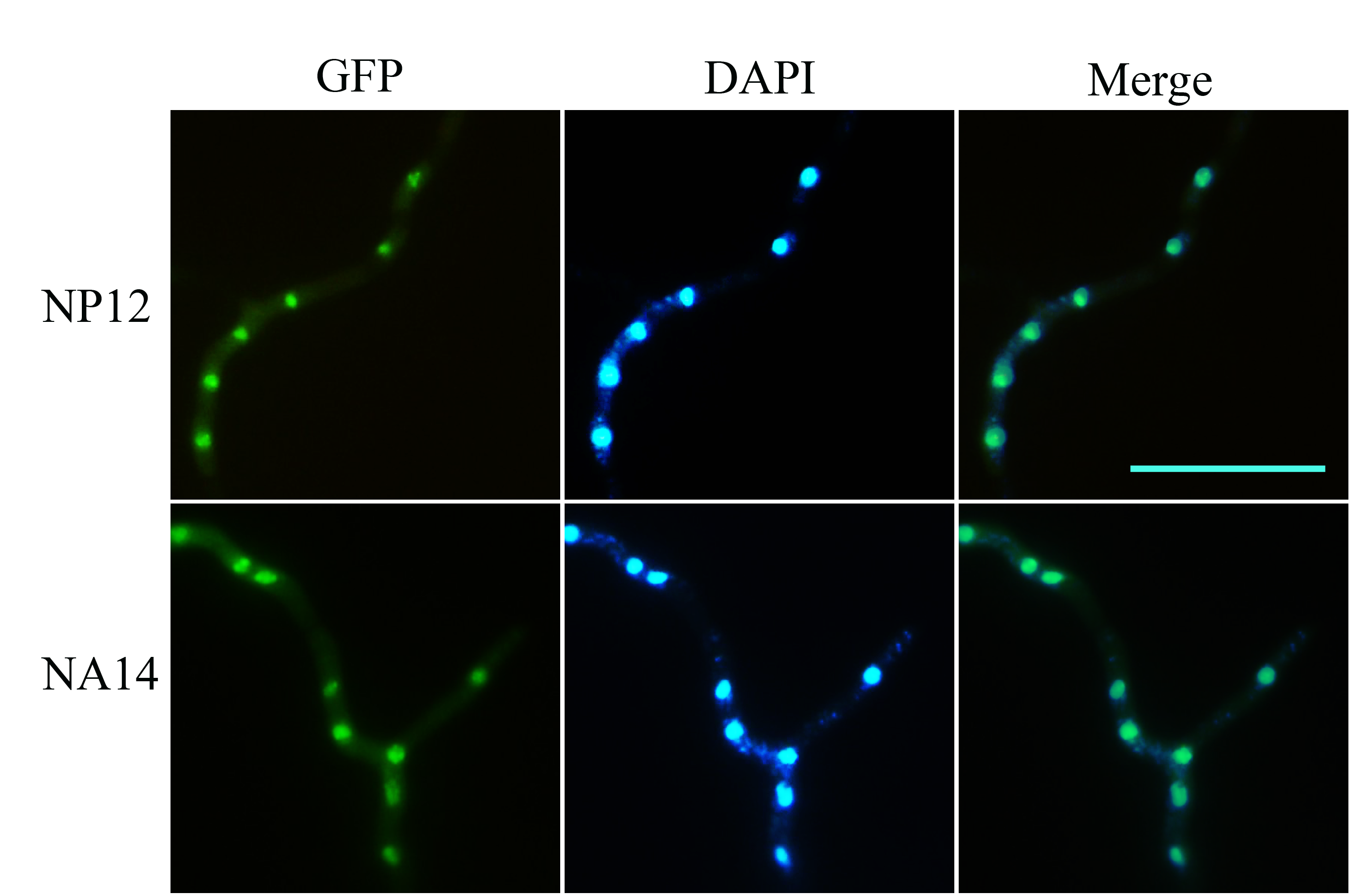

Supplement: Figure S4 — Deletion of AMT1 had no effects on the nucleo-cytoplasmic transport of FgNab2. In both transformants of PH-1 (NP12) and Δamt1 (NA14) mutant expressing the FgNAB2-GFP fusion construct, GFP signals mainly localized to the nucleus. Bar = 20 µm. (TIF) [file pone.0038324.s004.tif]

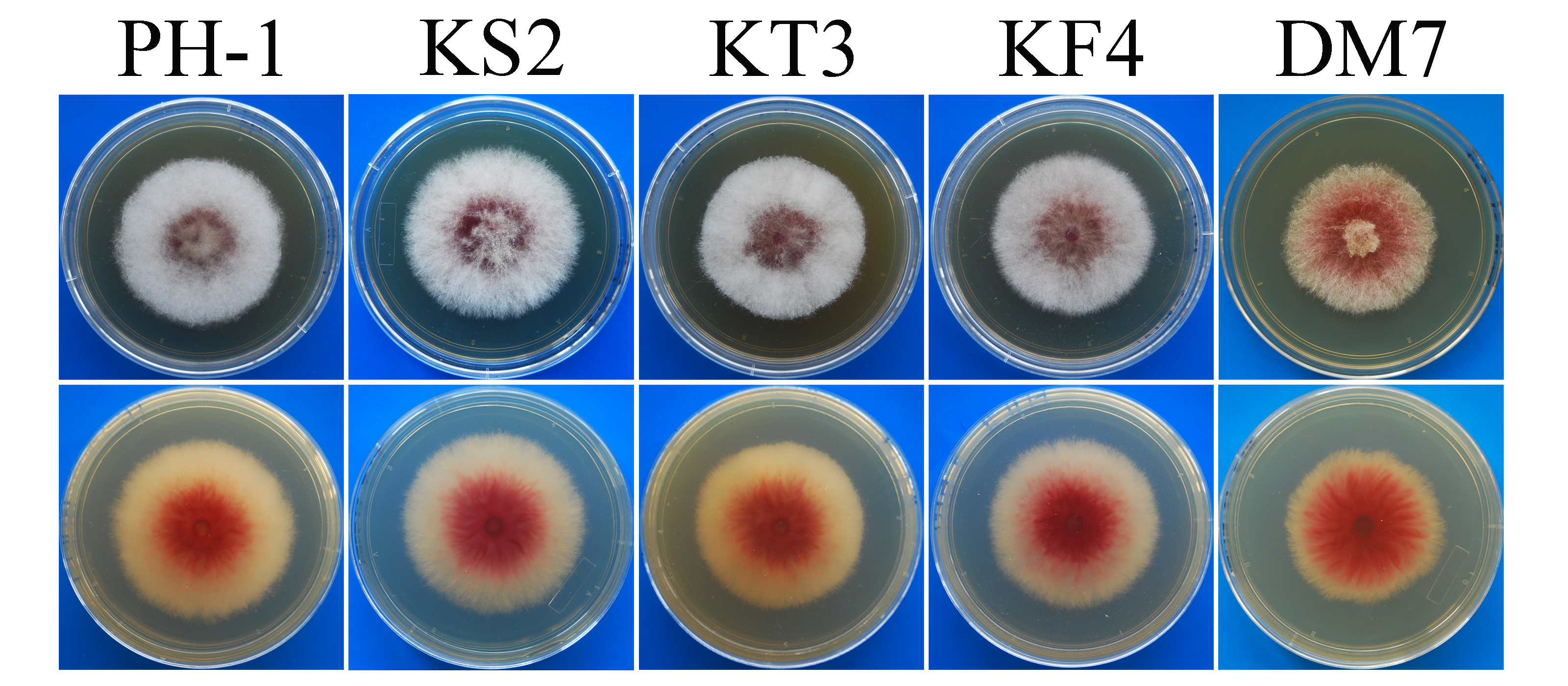

Supplement: Figure S5 — Three-day old PDA cultures of the wild-type strain (PH-1) and the Δ amt2 (KS2), Δ amt3 (KT3), Δ amt4 (KF4), and Δ amt1 Δ amt2 (DM7) mutants. (TIF) [file pone.0038324.s005.tif]

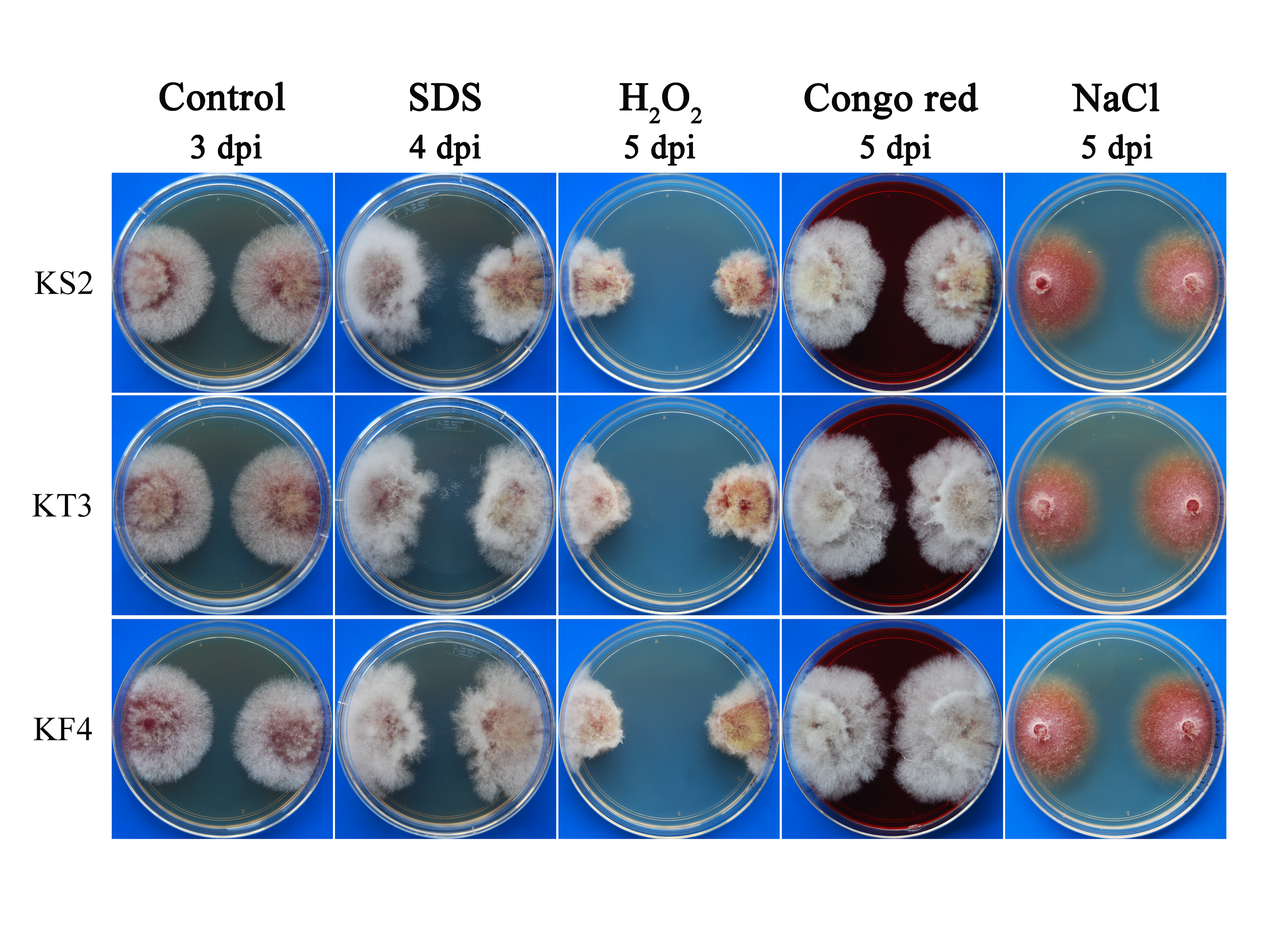

Supplement: Figure S6 — Assays for defects in stress responses. Cultures of the wild type (PH-1) and the Δamt2 (KS2), Δamt3 (KT3), and Δamt4 (KF4) mutants on PDA without or with 0.7 M NaCl, 300 µg/ml Congo red, 0.05% H2O2, or 0.01% SDS. Photographs were taken after incubation at 25°C for 3–5 days as labeled. (TIF) [file pone.0038324.s006.tif]
