# Supplementary material for: The AMT1 Arginine Methyltransferase Gene Is Important for Plant Infection and Normal Hyphal Growth in Fusarium graminearum
Source: PLoS One. 2012 May 31;7(5):e38324. doi: 10.1371/journal.pone.0038324 (PMC3365026; doi:10.1371/journal.pone.0038324)
Supplement: Table S1 — Disease index of AMTs mutants in the wheat head infection. (DOC) [file pone.0038324.s007.doc]

**Table S1. Disease index of AMTs mutants in the wheat head infection.**

| strains | Disease index |
| --- | --- |
| PH-1 (WT) | 13.8±3.8 |
| M2 (△*amt1*) | 4.3±3.7 |
| KS2 (△*amt2*) | 7.2±1.9 |
| KT3 (△*amt3*) | 8.2±6.6 |
| KF4 (△*amt4*) | 7.4±4.3 |
| DM7 (△*amt1*△*amt2*) | 4.2±1.4 |
| DM12 (△*amt1*△*amt2*) | 4.2±3.2 |
