# Supplementary material for: The AMT1 Arginine Methyltransferase Gene Is Important for Plant Infection and Normal Hyphal Growth in Fusarium graminearum
Source: PLoS One. 2012 May 31;7(5):e38324. doi: 10.1371/journal.pone.0038324 (PMC3365026; doi:10.1371/journal.pone.0038324)
Supplement: Table S2 — PCR primers used in this study. (DOC) [file pone.0038324.s008.doc]

**Table S2. PCR primers used in this study**

| **Name** | **Sequence (5’-3’)** |
| --- | --- |
| AMT1/1F | aggttgtttggggtcgctacgc |
| AMT1/2R | ttgacctccactagctccagccaagccccacagacgaatgcgattttgc |
| AMT1/3F | gaatagagtagatgccgaccgcgggttttcatagtcccgagagccccttt |
| AMT1/4R | atgacctacaatgacctccccaac |
| AMT1/5F | tctacgctcgtgacacctacctc |
| AMT1/6R | tacttggtgtggggacctgtt |
| AMT1/7F | cagaaccccctatacactca |
| AMT1/8R | ctacccgtgctttccgtgag |
| YG/F | gatgtaggagggcgtggatatgtcct |
| HY/R | gtattgaccgattccttgcggtccgaa |
| HYG/F | ggcttggctggagctagtggaggtcaa |
| HYG/R | aacccgcggtcggcatctactctattc |
| H852 | aactcaccgcgacgtctgtc |
| H850 | ttgtccgtcaggacattgtt |
| H855R | gctgatctgaccagttgc |
| H856F | gtcgatgcgacgcaatcgt |
| AMT1-CM/F | aactgcagaagaacaagcgagccga |
| AMT1-CM/R | cgggatccgcacagtttaacgatgaatg |
| AMT1-YA/F | cgactcactatagggcgaattgggtactcaaattggaagaacaagcgagccga |
| AMT1-YA/R | caccaccccggtgaacagctcctcgcccttgctcacgcacatgcggtattcgag |
| HRP1-pFL2/F | cagatcttggctttcgtaggaacccaatcttcaatgttcattggtggtctcaactggga |
| HRP1-pFL2/R | caccaccccggtgaacagctcctcgcccttgctcactcgtgagtaggggtggaatccg |
| NAB2-pFL2/F | cagatcttggctttcgtaggaacccaatcttcaatgcctgtcgaggtcagccttaac |
| NAB2-pFL2/R | caccaccccggtgaacagctcctcgcccttgctcactccgatgacatcagcctcttgct |
| Fg10718-1F | aagccagactcgcaagggtaag |
| Fg10718-2R | ttgacctccactagctccagccaagccggacgagactatgccctgttgg |
| Fg10718-3F | gaatagagtagatgccgaccgcgggtttgaggcaaggatgataagaggtgt |
| Fg10718-4R | gctgtggtgagtatggcaggatgt |
| Fg10718-5F | ttgccaattatcgcctga |
| Fg10718-6R | tcccgagcatacaacacg |
| Fg10718-7F | cgaggttgatttgatgaag |
| Fg10718-8R | atcctgtatccggaatcagcaaa |
| Fg00501-1F | gcagaaggcaactgacgaccaac |
| Fg00501-2R | ttgacctccactagctccagccaagccgctaacccacttgacaagacgca |
| Fg00501-3F | gaatagagtagatgccgaccgcgggtttgatgcctacctcacgcctttgctt |
| Fg00501-4R | ctctggatgcctccttcctgtctgc |
| Fg00501-5F | ccctcacgccaccacattatc |
| Fg00501-6R | ctgccccctcgaaggacttact |
| Fg00501-7F | ggttgcttacctggagatagt |
| Fg00501-8R | tgagggtggtagcattgtgagt |
| Fg10756-1F | gatggcaaagcgagaaggagtagg |
| Fg10756-2R | ttgacctccactagctccagccaagccacggtaggcagttggaataaatagg |
| Fg10756-3F | gaatagagtagatgccgaccgcgggttcccgactctgagatgaccaggataa |
| Fg10756-4R | aacatacagtctcctccaaatgccac |
| Fg10756-5F | cagcagcctccattcacctac |
| Fg10756-6R | acctgaccgctttgcttcg |
| Fg10756-7F | gccattgctatcaccgttgt |
| Fg10756-8R | accaatcagaaccctccaactca |
